# Acute cholangitis due to pancreatic metastasis from squamous cell lung carcinoma: a case report and review of literature

**DOI:** 10.1186/1757-1626-2-9113

**Published:** 2009-11-30

**Authors:** Maria A Kyriazi, Chrisostomos Sofoudis, Maria Katsouri, Theocharis Kappos, Christos Zafeiris, Eleni Trihia, Pantelis Diamantopoulos, Iakovos N Nomikos

**Affiliations:** 1 2nd Department of Surgery, "Metaxa" Cancer Memorial Hospital, Pireaus, Greece; 2 Department of Pathology, "Metaxa" Cancer Memorial Hospital, Pireaus, Greece

## Abstract

**Introduction:**

The pancreas is a well-documented but relatively uncommon site of non-small-cell cancer metastases. However, at the time of diagnosis the disease is usually locoregionally advanced, therefore therapeutic management is mostly palliative and symptomatic.

**Case Presentation:**

We report the case of a 77-year-old Caucasian male patient who presented initially with a clinical picture of acute cholangitis approximately 2 years after a left lower lobectomy for a low-grade squamous lung carcinoma. CT scan imaging of the abdomen and chest revealed an abnormal growth of the pancreatic head and distention of both the intra- and extra-hepatic billiary tree, whereas osteolytic abnormalities were observed of the 5th left rib, consistent with secondary deposits. Initially an endoscopic retrograde cholangio-pancreatography (ERCP) and sphincterectomy was performed and a plastic stent was placed in the common bile duct to decompress the biliary tree. Cytological examination of the aspirate collected by FNA of the pancreatic lession under EUS guidance revealed cells consistent with a low grade squamous lung carcinoma. Two months later an open cholecystectomy along with a gastrojejunostomy was performed to relieve the patient's gastric outlet obstruction symptoms. Following remission of the patient's attack of acute cholangitis and excessive vomiting he was released from the hospital and instructed to initiate chemotherapy with vinorelbine. The patient succumbed to disseminated disease almost 5 months later.

**Conclusion:**

Symptomatic metastatic lesions of the pancreas from squamous cell carcinoma of the lung are infrequent. Typically, the patients remain asymptomatic until their disease reaches a fairly advanced stage and therapeutic options are limited to palliative measures. A high index of suspicion is the only way of early detection and potentially effective treatment for this rare localization of metastatic squamous lung carcinoma.

## Introduction

A variety of malignant tumors have been documented to metastasize to the pancreas. The most common primary site for pancreatic metastases is the lung (18-27%) [1,2]. However, this relatively high occurrence of solitary pancreatic metastases from lung cancer is mainly based on autopsy reports. These tumors are usually asymptomatic or present with vague symptoms that can delay the diagnosis of metastatic disease. When they become clinically evident, their most common manifestations are that of obstructive jaundice and/or acute pancreatitis [2,3]. These cases, usually involve patients with widespread, disseminated disease, so therapeutic management is mostly palliative and symptomatic. Nonetheless, there have been a few scattered reports of radical surgical interventions to selected patients.

In this report, we present the case of 77-year-old patient with non-small-cell lung carcinoma who presented with a metachronous solitary pancreatic metastases that became clinically evident with recurring episodes of cholangitis and obstructive jaundice, as well as symptoms of gastric outlet obstruction.

## Case-Presentation

A 77-year-old Caucasian male patient, with a history of triscupid deficiency, coronary heart disease, arterial hypertension and a permanent pacemaker placement due to bradyarrythmia was diagnosed with a solitary lesion of the lower lobe of the left lung on September 2006, an incidental finding in a routine chest x-ray. This finding was confirmed by a chest CT, which in turn revealed a 2.5 × 2 cm solitary lesion on the lower lobe of the left lung. At that time, no evidence of metastatic disease was demonstrated from the patient's additional radiologic examination. Subsequently, he underwent a lower lobectomy of the left lung with an uneventful recovery.

Pathological examination confirmed a low-grade squamous carcinoma of the lung, with peripheral spots of adenocarcinoma with clear surgical margins and negative lymph nodes. Postoperatively the patient received a regime of adjuvant chemotherapy consisting of 4 cycles of Paclitaxel (Taxol) and Carboplatin.

**Figure 1:**
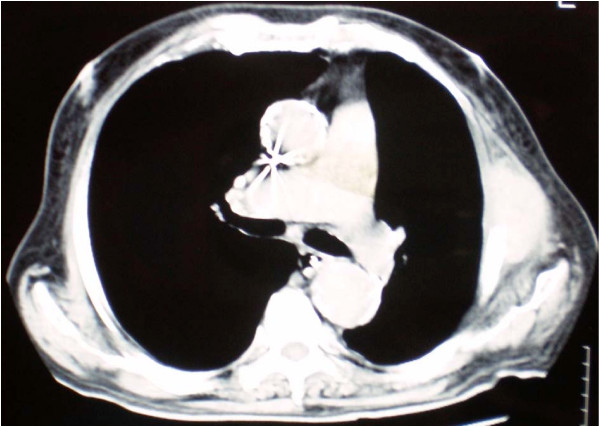
**CT scan image of the patient's chest showing osteolytic abnormalities of the 5^th ^left rib, consistent with secondary deposits**.

**Figure 2:**
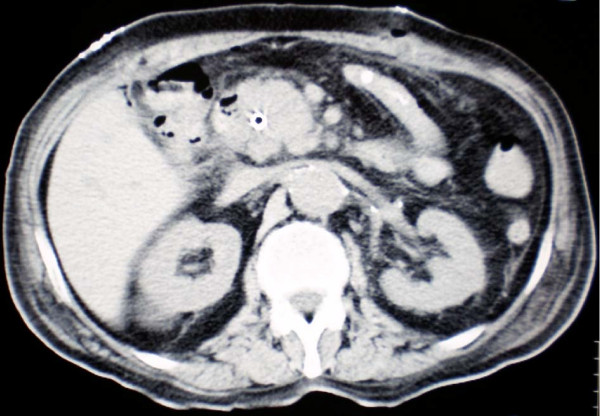
**Abdominal CT scan image showing the well circumscribed lesion of the pancreatic head (the common bile duct stent is clearly discerned)**.

**Figure 3:**
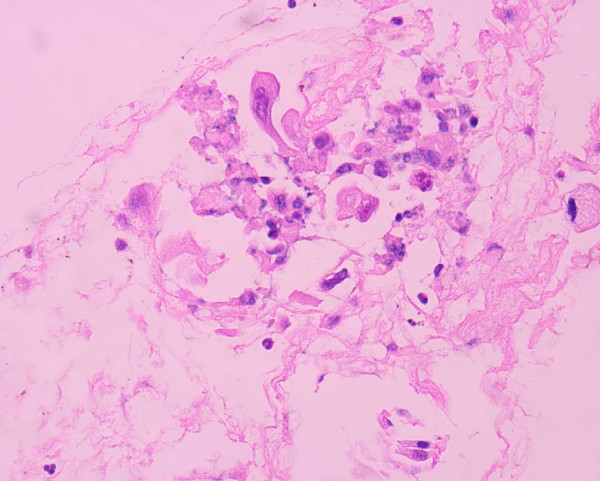
**View of cytological picture, demonstrating abnormal pancreatic cells, with squamoid features (H & E stain, 2 × 400)**.

**Figure 4:**
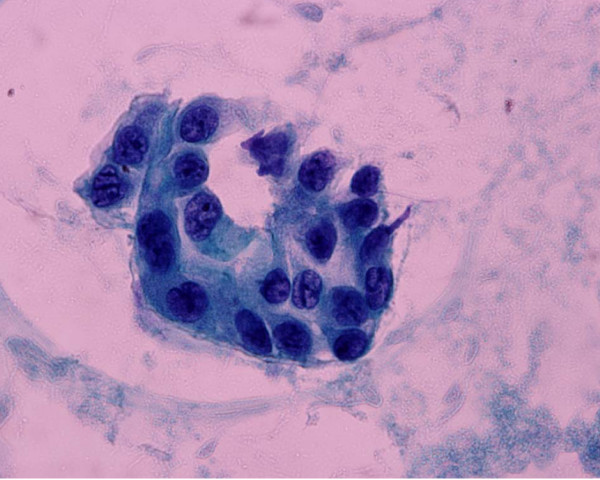
**View of cytological picture, demonstrating abnormal epithelial cells with subtle tubular arrangement (Pap stain, 4 × 600)**.

For the following 2 years the patient did well without any evidence of local or systemic recurrence. On November 2008 a routine follow-up chest CT revealed osteolytic abnormalities of the 5^th ^left rib, consistent with secondary deposits [Figure 1]. A few days later the patient was admitted to the hospital presenting with high fever (38.5°C), rigor, recurrent vomiting resulting in incapability to eat, right upper quadrant pain and jaundice. Radiological examination of the abdomen with both an ultrasound and a computed tomography revealed cholelithiasis and a highly suspicious, well circumscribed lesion of the pancreatic head, with both cystic and solid elements, resulting in distention of both intra- and extra-hepatic biliary tree and causing pyloric stenosis [Figure 2]. A protruding, distorted Vater ampulla with adenomatoid appearance, as well as distention of intra- and extra-hepatic billiary tree, secondary to stenosis of the distal common bile duct was revealed on a subsequent ERCP. Endoscopic sphincterectomy was performed and a plastic stent was placed in the common bile duct. Fine needle aspiration of the pancreatic head lesion under EUS guidance disclosed a low grade squamous carcinoma with immunohistochemical characteristics consistent with metastatic lung carcinoma [Figures 3, 4]. Two months later the patient underwent a gastrojejunostomy and an open cholecystectomy due to persistent symptoms of gastric outlet obstruction.

The patient had an uneventful recovery and remained in good clinical condition for the following three months. Progressively he developed symptoms of disseminated disease and finally died five months post-laparotomy.

## Discussion

The pancreas is a relatively infrequent site of distant metastasis. Moreover, metastatic tumors to the pancreas rarely become clinically evident, although their incidence has been reported to be approximately 12% in autopsy reports of patients suffering from other malignancies [4].

The most frequent sources of pancreatic malignant metastases originate from the lung, breast, kidney, gastrointestinal tract, thyroid, melanoma, and liver. Melanomas and osteosarcomas are also among the tumors that metastasize to the pancreas. The route of metastases is lymphatic (28%), vascular (27%), lymphatic - vascular (19%) and by direct invasion (18%) [1]. Such lesions usually appear in patients between 60-70 years of age [5]. The most common manifestation is that of a solitary mass, located in the head of the pancreas [5].

Primary lung cancer metastasizes to distant organs quite frequently. The most common sites of lung cancer metastases are the bones, liver and adrenal glands. The pancreas is a rather uncommon location of metastatic lung cancer. Evidence about it is based on scattered case reports in the literature that usually concern patients at an advanced stage of their disease, thus eligible only for palliative treatment. It is estimated that the incidence of secondary pancreatic deposits resulting from the various types of lung cancer range from 14.2% - 18. 2% [1,6]. The majority of these cases arise from small cell lung carcinomas (63%), and only rarely from adenocarcinoma (11.4%), large cell carcinoma (5.7%), squamous cell carcinoma (5.7%) and anaplastic carcinoma (2.9%) [7].

Symptoms caused by metastatic pancreatic lesions are variable and most patients are free of organ - specific complaints. Metastatic disease is usually incidentally detected on abdominal CT scan during the follow-up period. Those patients that do have clinical manifestations may present with abdominal or back pain, nausea, weight loss, jaundice, gastrointestinal haemorrhage or intestinal obstruction [8]. Moreover, whenever the pancreatic metastatic lesion directly invades the pancreatic duct epithelium it may clinically mimic primary pancreatic adenocarcinoma or, less commonly, induce acute pancreatitis [9-11].

The diagnosis is usually confirmed by percutaneous fine needle aspiration of the pancreatic lesion under CT guidance or endoscopic ultrasound (EUS) or by cytological examination of brushing specimens obtained during endoscopic retrograde cholangiopancreatography (ERCP)).

Treatment options for metastatic lung cancer lesions to the pancreas are mainly of palliative intent. They can be either non-invasive or invasine - surgical. Non-invasive treatment options can be chemotherapy and/or common bile duct stenting, in order to relieve the patient from jaundice and its symptoms. When surgical treatment is contemplated this is usually limited to by-pass procedures in patients with obstructive jaundice. There have been a few reports of patients who underwent pancreatic resections for metastatic lung cancer lesions, but this was either in ignorance of or overseeing the fact that the aetiology of the obstruction was of metastatic origin [3,12]. There have been several papers suggesting that pancreatectomy for metastatic lesions may result in improved survival rates and disease free intervals [13-15]. However, these results involve patients with metastatic pancreatic lesions of different histologic origin, such as renal cell cancer, lung, breast and colonic cancer. Further studies are required in order to determine whether aggressive surgical treatment is beneficial in patients with secondary pancreatic deposits from lung cancer.

Non-small cell lung cancer with distant metastases (stage IV) has a poor prognosis. Platinum-based chemotherapy regimens have been shown to improve survival and enhance quality of life, and they are also cost effective. This treatment is most appropriate for patients with a good performance status. EGFR inhibitors are used as second or third line therapy. They are most effective in women, in patients who have never smoked, or are diagnosed with adenocarcinoma. Studies of other novel agents and non-platinum-based regimens are ongoing. Median survival has been reported to improve from 3.6 to approximately 6.5 months after chemotherapy [3]. Resection of an isolated brain metastasis in patients with a good performance status has been shown to improve survival. However, there is very little information about the survival benefit resulting from resection of solitary metastasis to the pancreas with curative intent. In a small series by Hiotis et al of 3 patients with metachronous non-small-cell cancer pancreatic metastasis who underwent pancreatectomy, all patients were reported to have eventually developed recurrence [3,15]. Whether all patients who are at acceptable risk for surgery should be offered pancreatic resection for isolated mestatatic disease from lung cancer should be the subject of further future investigations.

Finally, the information above highlights the fact that a high index of suspicion should be raised for every patient with a previous history of cancer, who presents with a pancreatic mass. Therefore, before making any therapeutic decision, any correlation of the pancreatic mass with the patient's previous cancer history should be thoroughly examined.

## Conclusion

Symptomatic metastatic lesions of the pancreas from squamous cell carcinoma of the lung are extremely rare. Typically, the patients remain asymptomatic until their disease reaches a fairly advanced stage, and therapeutic options are then limited to palliative measures. FNA of the suspsicious lession is fundamental in order to achieve differential diagnosis from other primary pancreatic tumors. A high index of suspicion is the only way of early detection and potentially effective treatment for this rare localization of metastatic squamous lung carcinoma.

## Consent

Written informed consent was obtained from the patient for publication of this case report and accompanying images. A copy of the written consent is available for review by the journal's Editor-in-Chief.

## Competing interests

The authors declare that they have no competing interests.

## Authors' contributions

MAK: designed and drafted the manuscript. CS: participated in the acquisition of data and preparation of the manuscript. MK: responsible for critical revision of scientific content.

TK: assisted in the preparation of the manuscript. CZ: assisted in the preparation of the manuscript. ET: performed histopathological, immunohistochemical and cytological analysis.

PD: participated in the acquisition of data and preparation of the manuscript. INN: the surgeon, approved the final version of the manuscript for publication. All authors read and approved the final version of the manuscript.

## References

[B1] Nakamura E Shimizu M Itoh T Manabe T Secondary tumors of the pancreas: clinicopathological study of 103 autopsy cases of Japanese patients Pathol Int 2001 51 9 686 90 10.1046/j.1440-1827.2001.01258.x 11696171

[B2] Liratzopoulos N Efremidou EI Papageorgiou MS Romanidis K Minopoulos GJ Manolas KJ Extrahepatic biliary obstruction due to a solitary pancreatic metastasis of squamous cell lung carcinoma. Case report J Gastrointestin Liver Dis 2006 15 1 73 5 16680238

[B3] Pericleous S Mukherjee S Hutchins RR Lung adenocarcinoma presenting as obstructive jaundice: a case report and review of literature World J Surg Oncol 2008 6 120 19014447 10.1186/1477-7819-6-120 PMC2615008

[B4] Abrams HL Spiro R Golstein N Metastases in carcinoma: analysis of 1000 autopsied cases Cancer 1950 3 74 85 10.1002/1097-0142(1950)3:1<74::AID-CNCR2820030111>3.0.CO;2-7 15405683

[B5] Boudghène FP Deslandes PM LeBlanche AF Bigot JM US and CT imaging features of intrapancreatic metastases J Comput Assist Tomogr 1994 18 905 910 7962797 10.1097/00004728-199411000-00010

[B6] Mousa A Mitry E Hammel P Sauvent A Nassif T Palazzo L Malka D Delchier JC Buffet C Chaussade S Aparicio T Lasser P Rougier P Lesur G Pancreatic metastases: a multicentric study of 22 patients Gastroenterol Clin Bio 2004 28 872 876 10.1016/s0399-8320(04)95151-2 15523224

[B7] Maeno T Satoh H Ishikawa H Yamashita YT Fujiwara M Kamma H Ohtuka M Hasegawa S Patterns of pancreatic metastasis from lung cancer Anticancer Res 1998 18 2881 2884 9713480

[B8] Merkle EM Boaz T Kolokythas O Haaga JR Lewin JS Brambs HJ Metastases to the pancreas Br J Radiol 1998 71 851 1208 14 10434919 10.1259/bjr.71.851.10434919

[B9] Rumancik WM Megibow AJ Bosniak MA Hilton S Metastatic disease to the pancreas: evaluation by computed tomography J Comput Assist Tomogr 1984 8 5 829 34 10.1097/00004728-198410000-00003 6470248

[B10] Roland CF van Heerden JA Nonpancreatic primary tumors with metastasis to the pancreas Surg Gynecol Obstet 1989 168 4 345 7 2928909

[B11] Stewart KC Dickout WJ Urschel JD Metastasis-induced acute pancreatitis as the initial manifestation of bronchogenic carcinoma Chest 1993 104 1 98 100 10.1378/chest.104.1.98 8391965

[B12] Kotan C Er M Ozbay B Uzun K Barut I Ozgoren E Extrahepatic biliary obstruction caused by small-cell lung cancer: a case report Acta Chir Belg 2001 101 4 190 2 11680063

[B13] Minni F Casadei R Perenze B Greco VM Marrano N Margiotta A Marrano D Minni F Casadei R Perenze B Greco VM Marrano N Margiotta A Marrano D Pancreatic metastases: observations of three cases and review of the literature Pancreatology 2004 4 6 509 20 Epub 2004 Aug 16. 10.1159/000080248 15316227

[B14] Le Borgne J Partensky C Glemain P Dupas B de Kerviller B Pancreaticoduodenectomy for metastatic ampullary and pancreatic tumors Hepatogastroenterology 2000 47 32 540 4 10791233

[B15] Hiotis SP Klimstra DS Conlon KC Brennan MF Results after pancreatic resection for metastatic lesions Ann Surg Oncol 2002 9 7 675 9 10.1007/BF02574484 12167582

